# Scientific Items

**Published:** 1886-06-20

**Authors:** 


### SCIENTIFIC ITEMS.

**Inoculation as a Preservative against Consumption.**—M. Verneuli has lately published a letter to the editor of the *Gazette Hebdomidaire*, M. Lereboullet, in which he proposes to set on foot an experimental inquiry into the possibility of finding some method of "attenuating" the presumed virus of tubercle, so as to make inoculation therewith practically useful against consumption, either as a prophylactic measure, like vaccination against small-pox, or as a means of cure, like Pasteur's inoculations in hydrophobia.

Three thousand francs have already been subscribed, and the respectable names of Cornil, Bouchard, Damaschino, and Potain are mentioned among those who approve of the investigation.

It must, however, be remembered (1) that with the exception of hydrophobia, an exception still on trial, no human disease but small-pox is known which can be prevented by inoculation; (2) that of epizootic diseases, anthrax is only in certain cases guarded against by Pasteur's attenuated virus; (3) that the dependence of consumption on Koch's *Bacillus tuberculosis* is far from established; (4) that its fatality is very far below that of small-pox or hydrophobia, and its treatment far more successful.

Consumption is the most important disease of temperate climates, both by its prevalence, its mortality, and its incidence on young adults; so that the sacrifice of a few rabbits or cats for even a remote chance is, we fear, remote.—*Nature.*

**Photographing Celestial Bodies.**—It is now affirmed that many celestial objects which, by reason of the great refrangibility of their rays, would entirely escape our notice, can be discovered by photography.

"The solar photographs show numerous examples of this fact; and it was by them that my attention has been especially attracted to this point, which I have also had the opportunity of verifying by photographs of the stars. Thus, for example, in 1881–82 a photograph of the constellation of Orion showed with great distinctness many stars which were scarcely visible to the eye in my telescope of one-half meter [twenty inches] aperture. The Messrs. Henry have recently obtained proof of the actual existence of a nebula in the Pleiades, which, although photographed many times, has only lately been observed through the great thirty-inch object-glass of the Pulkowa observatory.

"The reason of this is, that the radiations from those bodies were richer in actinic or chemical rays than in luminous ones.

"In a note presented to the Academy on the 31st of December, 1877, and at other times, I have said that the photographic method not only offers a means of fixing and preserving luminous images, but that it constitutes a new means of scientific discovery, especially in astronomy. I added that the sensitive surface of the photographic plate, by reason of its power of recording the impress of the numerous rays that make no impression upon the human eye, should be considered as the true retina of the *savant.*'—*Pop. Science News.*

**The Wonders of an Egg.**—Mr. Matthieu Williams, in one of his lectures, says: "Every one who eats his matituatinal egg, eats a sermon and a miracle. Inside of that smooth, symmetrical, beautiful shell lurks a question which has been the Troy town for all the philosophers and scientists since Adam. Armed with the engines of war—the microscope, the scales, the offensive weapons of chemistry and reason—they have probed and weighed and experimented; and still the question is unsolved, the citadel unsacked. Professor Bokorny can tell you that albumen is composed of so many molecules of carbon and nitrogen and hydrogen, and can persuade you of the difference between active and passive albumen, and can show by wonderfully delicate experiments what the aldehydes have to do in the separation of gold, from his complicated solutions; but he can't tell you why from one egg comes a 'little rid hin,' and from another a bantam. You leave your little silver spoon an hour in your egg-cup, and it is coated with a compound of sulphur. Why is that sulphur there? Wonderful, that evolution should provide for the bones of the future hen! There is phosphorus also in that little microcosm; and the oxygen of the air, passing through the shell, unites with it, and the acid dissolves the shell, thus making good strong bones for the chick, and at the same time thinning the prison-walls. Chemists know a good deal now about albumen; and, if they cannot tell us why life differentiates itself therein and thereby, they can tell you how not to spoil your breakfast."—*Popular Science News.*

**Cattle Bones.**—The four feet of an ordinary ox will make a pint of neat's foot oil. Not a bone of any animal is thrown away. Many cattle's shin bones are shipped to England for the making of knife handles, where they bring $40 per ton. The thigh bones are the most valuable, being worth $80 per ton for cutting into tooth-brush handles. The fore-leg bones are worth $30 per ton and are made into collar buttons, parasol handles, and jewelry, though sheeps' legs are the staple parasol handles. The water in which the bones are boiled is reduced to glue, and the dust which comes from sawing the bones is fed to cattle and poultry.—*Scientific American.*

**The English "Parcels Post."**—The *Railway News* gives the new arrangements for the parcels post, which took effect May 1. The present maximum weight is seven pounds, which is to be increased to eleven pounds. The charges will be 3d. for the first pound and 1½d. for each pound or portion of a pound after the first, so that the charge for eleven pounds would be 18d., or 36 cents. There is also a provision for insurancc up to a value of £10, at an additional charge of 2d. Postal arrangements of this kind here would tend to diminish the value of express stocks.—*Ibid.*

